# Exogenous Insulin Injection-Induced Stiff-Person Syndrome in a Patient With Latent Autoimmune Diabetes: A Case Report and Literature Review

**DOI:** 10.3389/fendo.2020.00594

**Published:** 2020-09-02

**Authors:** Yi-Yin Lee, Cheng-Wei Lin, I-Wen Chen

**Affiliations:** Division of Endocrinology and Metabolism, Department of Internal Medicine, Chang Gung Memorial Hospital, Taoyuan City, Taiwan

**Keywords:** diabetes mellitus, autoimmune disease, Stiff-person syndrome, insulin, latent autoimmune diabetes in adults (LADA)

## Abstract

Stiff-person syndrome (SPS) is highly associated with anti-glutamic acid decarboxylase (GAD) antibody. However, GAD antibodies alone appear to be insufficient to cause SPS, and they possibly are involved in only part of its pathophysiology. It is suspected that the symptoms of SPS get precipitated by external stimuli. Here, we briefly introduce the case of a patient with latent autoimmune diabetes who developed SPS through the action of subcutaneously injected insulin. A 43-year-old man was diagnosed with diabetes and initially well-controlled with oral hypoglycemic agents but progressed to requiring insulin within 1 year of diagnosis. Two months after the initiation of basal insulin therapy, he presented with abdominal stiffness and painful muscle spasms, involving the lower limbs, which resulted in walking difficulty, and thus, he refused insulin injections thereafter. He had been treated with oral anti-diabetic agents instead of insulin for 10 years until premixed insulin twice daily was started again due to poor diabetes control. Immediately after insulin injection, abdominal muscle rigidity and spasms were noted. When insulin was not administered, frequent episodes of diabetic ketoacidosis occurred. Serum GAD antibody test was positive and there was no positivity for islet antigen-2 antibody. A glucagon stimulation test demonstrated relative insulin deficiency, indicative of latent autoimmune diabetes in adults (LADA). Tolerable muscle rigidity was achieved when the dosage of basal insulin was split into two separate daily injections with lower amounts of units per injection. This case highlights a different form of autoimmune diabetes in SPS. To our knowledge, this is the first report of SPS described shortly after the initiation of insulin therapy that required basal insulin to achieve tolerable muscle symptoms and better glucose control, without the development of diabetic ketoacidosis.

## Introduction

Stiff-person syndrome (SPS) is a rare autoimmune neurological disorder and highly associated with glutamic acid decarboxylase antibody (GAD-Ab) positivity. Gamma aminobutyric acid (GABA) is the predominant inhibitory neurotransmitter in the central nervous system. In the neuron, GABA is synthesized in the cytosol from its precursor glutamate by the enzyme glutamate decarboxylase (GAD) and then transported into the synaptic vesicles (1–3). The blockade of GAD, a critical enzyme involved in inhibitory pathways, and subsequent decline in the levels of GABA in the central nervous system have been suggested to be associated with a loss of neural inhibition, although evidence for a causative link between GAD antibodies and SPS pathogenesis is still lacking (1). GAD is the rate-limiting enzyme for the synthesis of GABA. SPS is characterized by progressive muscle stiffness, rigidity, and spasm involving the truncal and proximal limb muscles, resulting in severely impaired ambulation (4). It is suspected that the symptoms of SPS get precipitated by external stimuli, such as sounds, stress, and emotional upheaval (1, 5). The diagnosis of SPS is based on the detection of typical clinical findings, GAD antibody positivity, and suggestive ancillary investigations. GAD is also found in non-neuronal tissues, such as pancreatic beta cells, testes, and oviducts, so SPS is also associated with other endocrine disorders (6, 7). It has been shown that ~45% of patients with SPS have type 1 diabetes (T1D), which is an autoimmune disease characterized by the immune-mediated loss of the insulin-producing pancreatic beta cells (1, 5, 8, 9). SPS and T1D share human leukocyte antigen (HLA) genetic risk, with most SPS patients possessing the DQ2 phenotype (10). Latent autoimmune diabetes in adults (LADA) shares clinical and metabolic characteristics with both T1D and type 2 diabetes (T2D). According to the criteria of LADA diagnosis, LADA is defined by the absence of insulin requirement for at least 6 months after diagnosis and exhibits an older age of onset, as well as a slower progression toward insulin dependence compared to subjects with T1D (11, 12). LADA in SPS is rarely described (13). Here, we reported a case of SPS in a patient with LADA. To our knowledge, this is the first report of SPS described shortly after the initiation of insulin therapy that required to split the administration of basal insulin into two separate injections (with lower amounts of insulin units per each injection) to achieve tolerable muscle symptoms and better glucose control, without the development of diabetic ketoacidosis.

## Case Report

A 43-year-old man with body mass index (BMI) 28.2 kg/m^2^, diagnosed with diabetes mellitus in 2005 with a family history of T2D (parents, one uncle, and one sibling) presented with thirst and weight loss and was initially administered oral anti-diabetic drugs (metformin 1,500 mg/day). However, within 1 year, he was started on subcutaneous insulin injections (Insulatard, isophane Neutral Protamine Hagedorn human insulin, twice daily). Two months after the initiation of insulin therapy, he presented with abdominal stiffness, painful spasms extending to lower limbs, and impaired walking ability. Neurological examination showed muscle rigidity and bilateral contractures in the lower limbs but no muscular weakness. Electroencephalography was normal, and electromyography/nerve conduction velocity revealed slightly asymmetrical motor and sensory polyneuropathy with demyelination. These features were highly indicative of SPS. Therefore, diazepam (10 mg/day in two divided oral doses), clonazepam (2 mg orally four times daily), baclofen (10 mg two times daily), and phenobarbital (30 mg two times daily) were administered to treat muscle rigidity.

The patient's symptoms of jerky myoclonus-like movement, abdominal stiffness with painful spasms, and muscle rigidity were exacerbated by sound, anxiety, and subcutaneous insulin injections, regardless of the treatment regimen (Insulatard or regular insulin). Thus, he refused insulin therapy, resulting in poor glycemic control for 10 years, which resulted in glycated hemoglobin (HbA1c) values persistently >8.0% (64 mmol/mol) with only oral anti-diabetic drugs (metformin 2,000 mg/day, sitagliptin 100 mg/day, glimepiride 8 mg/day, and acarbose 200 mg/day). Therefore, basal insulin (insulin glargine, 0.43 IU/Kg/day) with oral anti-diabetic agents (metformin 1,500 mg/day and gliclazide MR 240 mg/day) followed by premixed insulin twice daily (Humalog Mix 25, 0.71 IU/Kg/day) or a basal-bolus injection regimen (insulin detemir 0.17 IU/Kg/day and insulin aspart 0.13 IU/Kg/day) were introduced since 2015. He consistently experienced muscle rigidity with subcutaneous insulin injections every time. His fear and anxiety of re-experiencing muscle stiffness resulted in poor compliance with the prescribed therapeutic regimens. Finally, he experienced two episodes of diabetic ketoacidosis in 2017 (BMI was 22.45 kg/m^2^) and the laboratory results are summarized in Table 1. His GAD65-Ab level was positive (110 units/mL), insulin antibodies (IAs) were positive (13.7%), while islet antigen-2 (IA2) autoantibodies were within the normal range. A thyroid function test revealed euthyroidism, and antithyroid peroxidase Abs were positive (18.81 IU/mL). A 6 min-glucagon stimulation test was performed under stable clinical conditions and demonstrated insulin deficiency (basal C-peptide levels, 0.26 ng/mL; stimulated C-peptide levels 0.41 ng/mL), which was compatible with a diagnosis of LADA.

**Table 1:** Results of laboratory tests.

**Variable**	**January 2017 diabetic ketoacidosis**	**April 2017 diabetic ketoacidosis**	**August 2017 outpatient clinic**	**Reference range**
Osmolality, msom/KgH_2_O	313	323		275–295
Blood ketone, mmol/L	5.2	5.1		<0.6
pH	7.274	7.264		7.31–7.41
Absolute base excess	−10.7	−10.7		
PCO_2_, mmHg	35.6	34.4		41–57
HCO_3_, mmol/L	16.4	15.7		22–26 (male)
Fasting glucose			272	
Fasting C-peptide, ng/mL	0.48		0.26	0.9–4.3
Glucagon-stimulated C-peptide, ng/mL			0.41	
HbA1c, %	13.1	13.2	13.4	
Insulin, mU/mL			1.0	1.5–17
GAD65-Ab, U/mL	110		110	<1
IA2-Ab, U/mL	0.65		0.31	<1
Insulin antibody (%)	13.7			<10%
TSH, mU/L	0.985	1.176		0.35–5.50
Free T4, ng/dL	1.11	1.18		0.76–1.64
Anti-TPO Ab, IU/mL			18.81	<5.6
Cortisol, g/dL	24.45		14.08	5–23

*HbA1c, glycated hemoglobin; GAD65-Ab, glutamic acid decarboxylase-65 antibody; IA2-A, islet antigen-2 antibody; TSH, thyroid stimulating hormone; Anti-TPO Ab, anti-thyroid peroxidase antibody*.

Intravenous immunoglobulin (IVIG, 0.4 g/kg/day) led to a poor response, but three courses of plasmapheresis improved the patient's clinical symptoms. Along with diazepam (20 mg in four separate oral doses) and clonazepam (2 mg four times daily), premixed insulin (Novomix 30, 0.46 IU/Kg/day), followed by basal insulin (insulin glargine U-300, 0.29 IU/Kg/day) with oral anti-diabetic drugs (sitagliptin 100 mg, pioglitazone 30 mg, metformin 1,700 mg, and gliclazide MR 120 mg) were administrated but muscle rigidity persisted after insulin injections every time. We splitted the administration of basal insulin into two separate injections (insulin degludec, 0.34 IU/Kg/day, injected twice daily, with a time interval between the two injections of 12 h) and oral hypoglycemic agents (linagliptin 5 mg, pioglitazone 30 mg, metformin 1,700 mg, and gliclazide MR 120 mg) were administered to prevent diabetic ketoacidosis, as the patient had subsequent tolerable abdominal stiffness.

## Discussion

SPS usually affects people in the age range of 30–60 years with an estimated prevalence of 1–2 cases per million individuals (1, 5). The presence of high-titers of GAD autoantibodies has been reported in more than 85% of patients with SPS (1, 14, 15). GAD is involved in the synthesis of the inhibitory neurotransmitter gamma aminobutyric acid in the central nervous system (CNS). Dysfunction of GABA input in motor neurons causes stiffness, rigidity, and painful spasms (16). GABAergic neuron dysfunction has also been reported in epilepsy, and 10% of patients with SPS have some form of epilepsy (1, 16). From preclinical and animal models, it has been hypothesized that autoimmune responses against GAD in pancreatic islet deplete this enzyme and reduce GABA levels within the GABAergic system in the endocrine pancreas, thus promoting the progression of T1D. Moreover, GABA has been shown to exert β-cell regenerative effects, protecting β-cells against apoptosis induced by cytokines, drugs, and other stresses, and exerting anti-inflammatory and immunoregulatory effects (17). The GAD-Abs are believed to play a role in the possible relationship between epilepsy and T1D (18). Two major isoforms of GAD enzyme exist, namely GAD65, and GAD67, which catalyze the formation of GABA at different locations in the cell and different time periods of development. GAD65 is attached to the surface of synaptic vesicles in GABAergic neurons or microvesicles in the pancreatic beta-cells, while GAD67 is a soluble form detectable only in the CNS (19). Anti-GAD antibodies are detected in up to 80% of SPS patients, while antibodies against the GAD67 isoform occur in <50% of patients and at much lower levels (19, 20).

SPS is characterized by fluctuating stiffness that affects truncal and proximal limb muscles, which progresses to the proximal lower limbs. The clinical features and differential diagnosis are listed in Tables 2, 3. GAD autoantibodies are also detected in disease other than SPS and autoimmune diabetes, such as epilepsy, cerebellar ataxia, myoclonus, myasthenia gravis, or other neurological disorders (16, 21). Regarding clinical manifestations, painful spasms are also observed as part of neuroleptic malignant syndrome and painful rigidity is also observed with tetanus (6). These symptoms can be induced by external stimuli, such as sounds, stress, and emotional upheaval (5). Low levels of GABA also lead to other neurological abnormalities, such as depression, panic disorders, and agoraphobia (5, 16, 22). The diagnostic approach and Dalakas' diagnostic criteria are summarized in Table 4 (1). Patients show variants that lack classical truncal and proximal limb distribution of stiffness and rigidity. Our patient experienced muscle rigidity and painful spasms after injection of a rapid-acting insulin analog, premixed insulin, or basal insulin. This is the first case of SPS described shortly after the initiation of insulin therapy. Although a rare event with human insulin and insulin analog, immunological response to exogenous insulin can occur (23). Antibodies to insulin are of two types, insulin antibodies (IAs) which occur in patients who have been exposed to exogenous insulin treatment, and insulin autoantibodies (IAAs) which appear in insulin-naïve subjects (24). Current human insulin and insulin analog therapies have resulted in decreased insulin antibodies (IAs) levels when contrasted with animal insulin (23). In the present case, although the patient used human insulin and insulin analogs rather than animal insulin as insulin therapy, IAs were positive. This may suggest the potential involvement of IAs in SPS pathogenesis beyond the stimulus of insulin injection. The association of SPS and immunological response to exogenous insulin requires further prospective studies to be validated.

**Table 2:** Main clinical features of Stiff-person syndrome.

✓ Insidious onset of symptoms ✓ Rigidity and tightness in truncal muscles (neck, paraspinal, and abdominal muscles) ✓ Painful spasms with disability ✓ Lumbar hyperlordosis is a diagnostic hallmark ✓ Facial muscle involvement is rare, lead to “emotionless mask” appearance ✓Triggered by sudden not well identified external stimuli ✓ Frequent coexisting psychiatric symptoms: task-specific phobias, depression, and generalized anxiety disorders ✓ Possible coexistence of other autoimmune diseases

**Table 3:** Differential diagnosis of Stiff-person syndrome.

**Differential diagnosis driven by GAD^†^ autoantibody positivity**
✓ Cerebellar ataxia ✓ Epilepsy ✓ Myoclonus ✓ Neuromyotonia ✓ Batten's disease ✓ Limbic encephalopathy ✓ Myasthenia gravis
**Differential diagnosis driven by clinical features**
✓Myelopathy ✓ Tetanus ✓ Neuromyotonia ✓ Parkinsonism ✓ Primary lateral sclerosis ✓ Ankylosing spondylitis ✓ Leukodystrophies ✓ Neuroleptic malignant syndrome

*GAD, glutamic acid decarboxylase*.

**Table 4:** Diagnostic approach and diagnostic criteria of Stiff-person syndrome.

**Diagnostic approach**
✓ Blood test: include complete blood count, electrolytes, thyroid function test, liver function test, fasting glucose, and HbA1c
✓ Oral glucose tolerance test
✓ Serum anti-GAD antibody ∙ (If anti-GAD ab negative, check anti-gephyrin and anti-amphiphysin, screening for paraneoplastic SPS)✓ Imaging study: MRI of brain or spinal cord, chest X ray, CT scan of chest, abdomen, or pelvis to exclude primary tumor ✓ Electromyography
**Dalakas' diagnostic criteria**
✓ SPS characterized by progressive muscle stiffness, rigidity, and spasm involving the truncal and proximal limb muscles, resulting in severely impaired ambulation ✓ Precipitated by sudden movement, noise, or emotional upset ✓ Confirmation of clinical and electromyography for continuous co-contraction of agonist and antagonist muscles, confirmed clinically, and electrophysiologically ✓ Absence of other neurological disorder that could lead to rigidity and stiffness ✓ Presence of GAD-65 autoantibody assessed by immunocytochemistry, radioimmunoassay (RIA), or Western blot ✓ Response to diazepam treatment

*HbA1c, glycated hemoglobin; GAD, glutamic acid decarboxylase; SPS, stiff-person syndrome; MRI, magnetic resonance imaging; CT, computed tomography*.

Hypoglycemia, weight gain, and injection site reactions are well-known possible complications of insulin therapy, which might interfere with a patient's willingness to start or continue insulin therapy or long-term adherence to insulin therapy. The most common neurological complications associated with insulin therapy are those related to insulin-induced hypoglycemia, such as confusion, blurred vision, and, in extreme cases, epilepsy, and coma (25–27). Ballout et al. reported the case of a 56-year-old man with T2D exhibiting painful cramps over the upper and lower extremities that occurred right after the subcutaneous injection of a rapid-acting insulin analog (insulin aspart). These symptoms were accompanied by a rapid drop in serum potassium levels that occurred shortly after the insulin injection. The subsequent symptom resolution occurring rapidly upon potassium supplementation suggested the insulin-induced drop in serum potassium levels as the likely cause of cramps (28). The previous findings clearly differed from those observed in our patient, who did not experience hypokalemia but showed stiffness of truncal muscles (while extremities were spared) upon insulin injections, sound, stress, or anxiety.

Insulin resistance and impaired insulin absorption from subcutaneous space due to dermal tension and stiffness in SPS was reported in a patient with T2D (29). Muscle tension, stiffness, and the additional induration of cutaneous and subcutaneous tissue further impaired insulin delivery via the subcutaneous routes causing catheter dysfunction in insulin pump, which did not allow for a needle longer than 8 mm and a higher amount of insulin to be applied per delivery (29). In contrast, our patient had experienced painful abdominal muscle spasms shortly after injection of a rapid-acting insulin analog, premixed insulin, or higher-dose of basal insulin. Tolerable muscle rigidity was achieved when reducing the daily dose of insulin and splitting the administration of basal insulin into two separate injections (with lower amounts of insulin units per each injection). These finding suggest that exogenous insulin-related immune response and/or higher insulin units and injection volumes may have contributed to the development of SPS shortly after the initiation of insulin therapy in our case.

While GAD-Abs, IA2-Abs, and IAAs are immunological biomarkers for T1D, an association of SPS with T1D is frequently observed in ~45% of patients with SPS (1, 5, 9, 30). GAD-Abs are detected in 80% of patients with newly diagnosed T1D, but the role of autoimmunity against GAD in the pathogenesis of SPS and T1D remains unclear. GAD-Abs are found in more than 85% of patients with SPS (1, 5, 14–16), and the GAD-Ab titer in these patients is much higher (around 50- to 100-fold) than that in patients with T1D alone (16). Anti-GAD65 autoantibodies in T1D are predominantly directed to conformational epitopes and the antibodies in SPS mostly recognize linear and denatured epitopes especially in the amino terminal region of the GAD65 molecule (1, 8).

LADA is a slowly progressive form of autoimmune DM, which is rarely reported concomitant with SPS (13). Because β-cell function is lost more gradually than in T1D but more rapidly than in T2D, patients may initially respond to non-insulin glucose-lowering agents. However, once β-cell function declines, their responses to these agents will diminish. LADA patients may initially respond to oral medications, and they often require insulin therapy within 3 years of diagnosis (31). According to the criteria of LADA diagnosis, LADA is defined by the absence of insulin requirement for at least 6 months after diagnosis (12). GAD-Abs are most frequently used for assessment of autoantibodies as a marker of the autoimmune activity that distinguish LADA from T2D due to the fact that these antibodies are far more common in patients with adult onset autoimmune diabetes than other autoantibodies often found in children with T1D (32, 33). Compared with those with low GAD-Ab titers, patients with high GAD-Ab titers (>32 arbitrary units) exhibit more prominent traits of insulin deficiency and a profile of more severe autoimmunity (34, 35). Patterns of susceptibility at the HLA-DRB1 and HLA-DQB1 loci in LADA are similar to those reported for T1D (36). Fourlanos et al. reported two cases of LADA patients diagnosed with SPS: at SPS diagnosis, one patient was on sulfonylurea, whereas the other was on insulin therapy since several years (13). Both two patients had class II HLA DQ2,8 phenotypes consistent with autoimmune diabetes. In our patient, diabetes was diagnosed 2 years prior to the diagnosis of SPS, and SPS was diagnosed shortly after the initiation of insulin therapy. Insulin was not required initially, but rapidly progressed to the requirement of insulin within 1 year of diabetes diagnosis. Two episodes of diabetic ketoacidosis with a positive GAD65-Ab test in 2017 and marginal residual beta-cell function detected upon glucagon stimulation test confirmed the diagnosis of LADA. However, we assessed HLA genotyping of patient that revealed HLA-DRB1^*^08; HLA-DRB1^*^13; and HLA-DQB1^*^06. Our results suggest HLA-DRB1 and HLA-DQB1 loci (which are involved in the genetic susceptibility to T1D) as important genetic risk factors for LADA.

Around 5–10% of patients with SPS have autoimmune thyroid disease (AITD), and the prevalence of GAD Abs is much higher in patients with AITD than in the general population (37). In addition to AITD (Graves' disease and Hashimoto's thyroiditis), SPS is also associated with pernicious anemia, vitiligo, and celiac disease (1, 38, 39).

Treatment of SPS is aimed at alleviating symptoms to improve daily activity and prevent disability. Therapeutic approaches include initial symptomatic treatments, such as muscle relaxants [first-line treatment involves the use of GABA A receptor agonists (e.g., benzodiazepines) and second-line treatment involves GABA B receptor agonists (e.g., baclofen)] and immunomodulatory therapy for refractory or severe SPS, including intravenous immunoglobulin, plasmapheresis, and B-cell-targeted therapy with rituximab (5, 16).

Anti-GAD Abs appear to be associated with the pathogenesis of SPS (although evidence of a causative link is still missing) and represent the immunological hallmark of autoimmune diabetes. Clinical suspicion and detection of anti-GAD Abs are important for the diagnosis of SPS. Early recognition and appropriate treatment are paramount to prevent long-term disability. Furthermore, physicians should keep in mind the association of SPS with autoimmune diseases, such as autoimmune thyroiditis, pernicious anemia, and autoimmune diabetes (T1D and LADA).

## Conclusion

In conclusion, we presented a case of SPS in a patient with LADA. To our knowledge, this is the first case reporting a dilemma wherein the patient required insulin therapy to achieve good glucose control and prevent acute and chronic complications, but developed SPS shortly after the initiation of insulin therapy.

## Ethics Statement

Written informed consent was obtained from the patients for the publication of this case report and related data in this article.

## Author Contributions

Y-YL was responsible for the literature search and review, case review, summary, and for drafting the original article. C-WL was responsible for article conception and literature review. I-WC was responsible for patient management and critical revision of the manuscript for intellectual content. All authors approved the final version and contributed significantly.

## Conflict of Interest

The authors declare that the research was conducted in the absence of any commercial or financial relationships that could be construed as a potential conflict of interest.
